# Small gifts, big shifts? Testing the role of contact through reciprocal gifting as a prejudice reduction strategy

**DOI:** 10.1111/bjso.70052

**Published:** 2026-02-01

**Authors:** Sami Çoksan, Mustafa Tercan, Sabahat Çiğdem Bağcı, Serpil Yıldız‐Çoksan

**Affiliations:** ^1^ Network for Economic and Social Trends Western University London Ontario Canada; ^2^ Department of Psychology, Faculty of Letters Erzurum Technical University Erzurum Turkey; ^3^ Republic of Türkiye Ministry of National Education Ankara Turkey; ^4^ Faculty of Arts and Social Science Sabanci University Istanbul Turkey; ^5^ Graduate School of Health Science, Child Development Ankara University Ankara Turkey

**Keywords:** attitudes, indirect contact, intergroup contact, intervention, reciprocal gifting, refugees

## Abstract

Through two experimental studies (pre‐test/post‐test/follow‐up with control), we tested reciprocal gifting as an indirect contact strategy that could improve Turkish native children's attitudes towards their Syrian refugee peers in the highly prejudicial immigration context of Türkiye. In Study 1 (*N* = 144), children who were led to believe that they exchanged gifts with their Syrian peers showed more positive outgroup attitudes in the post‐test (unlike children in the control group), while there were no significant changes in negative attitudes or social closeness. In Study 2 (*N* = 207), we implemented an enhanced procedure whereby children created personalized and symbolic gifts, making the reciprocal gifting experience more engaging. Although this revised approach improved positive attitudes and social closeness, negative attitudes remained unchanged, and all outcomes returned to baseline levels at the follow‐up stage (approximately 40 days later) in both studies, overall providing evidence for the short‐term positive effects of the reciprocal gifting strategy. We discussed the importance of implementing creative strategies in hostile school environments.

## BACKGROUND

According to the latest data from the United Nations High Commissioner for Refugees (UNHCR), approximately 122 million people have been displaced for various reasons, with around 4 million under the mandate of UNHCR (2024), highlighting the unprecedented scale of this migration in human history. This recent wave has led Türkiye to become the country hosting the largest number of refugees in the world over the past 5 years, with approximately 3 million registered Syrian refugees who have sought refuge in the country due to the Syrian Civil War. Throughout this period, many Syrian refugees have settled or obtained citizenship, securing permanent residence, as well as access to education in schools. However, the othering of refugees as a political tool by various components of the current political landscape (Sakarya, 2025), combined with citizens' perception of symbolic threat (the feeling that one's social identity, group values and beliefs are under attack), as well as realistic threat (competition over physical and material resources), has ultimately led to the development of hostile anti‐refugee attitudes and behaviours over time (e.g. Bagci et al., 2023; Çirakoğlu et al., 2021; Çoksan et al., 2023; Demir & Ozgul, 2019; Eren & Çavuşoğlu, 2021). In some regions, these behaviours have even escalated into acts of aggression (BBC, 2021) and calls for pogroms (Körükmez, 2025), which highlights the urgent need to devise effective strategies to combat the marginalization of refugees.

This negative treatment is particularly prevalent in state schools, where Syrian children report high levels of rejection and marginalization, alienation, as well as race‐based bullying (Çelik & İçduygu, 2019), and suffer from problems of integration in classrooms (Demir & Ozgul, 2019; Kilic & Gokce, 2020). Syrian children are often portrayed negatively by both their teachers and peers in school settings (Eren & Çavuşoğlu, 2021), and intergroup tensions between native and refugee children tend to increase over time, demonstrated by growing negative intergroup interactions and avoidance tendencies during the school year (Bagci et al., 2023). Overall, these findings underscore the necessity of implementing interventions aimed at enhancing positive relationships between native and refugee children in school settings. Motivated by this need, the current set of studies sought to improve local children's attitudes towards their Syrian peers by testing a structured indirect contact intervention based on the reciprocal gifting procedure, an approach rooted in Marcel Mauss' (1967) renowned anthropological studies. Combining intergroup contact and prosocial behaviour literatures, we conceptualized contact through reciprocal gifting as an indirect intergroup interaction mediated by the exchange of small objects with shared symbolic value, which operates around the obligations of *giving, receiving* and *reciprocating*. This interaction makes the intention to build a positive relationship visible by activating feelings of recognition and closeness between groups. Our procedure adapts this mechanism to an intergroup context by leading Turkish children to believe that they received gifts from Syrian peers and, in turn, were asked to reciprocate with similar gifts, thus creating a potential form of indirect intergroup contact that occurs through the experience of *prosocial exchange*.

### Intergroup contact and various forms of contact interventions

Intergroup contact refers to the experience of interaction between members of different social groups (Allport, 1954). It has been considered one of the key determinants of intergroup attitudes since the seminal works of Allport (1954) and Pettigrew (1998). Further meta‐analytical findings have consistently demonstrated that positive contact experiences lead to more positive intergroup relations across various intergroup contexts (e.g. Pettigrew & Tropp, 2006), mainly through reduced intergroup anxiety and increased outgroup empathy (Pettigrew & Tropp, 2008; Swart et al., 2011). Nevertheless, despite its pivotal role in improving intergroup harmony, contact opportunities are not always embraced (Paolini et al., 2018), and the natural formation of direct intergroup contact involves physical and psychological barriers such as segregation (e.g. Dixon et al., 2020), initial intergroup anxieties and threats, as well as norms that discourage contact in real‐life settings (e.g. Turner et al., 2007; Turner & Crisp, 2010). More importantly, even when contact opportunities exist, contact experiences may be negative and contribute to more destructive intergroup relationships (e.g. Kotzur & Wagner, 2021; Schäfer et al., 2021), creating a complex picture as regards the associations between intergroup contact and attitudes.

Especially when contact experiences are not pursued or include negativities, structured contact interventions could be implemented as a prejudice‐reduction strategy. Indeed, contact‐based interventions were argued to stand as one of the best strategies to reduce prejudice and negative attitudes (e.g. Lemmer & Wagner, 2015; Paluck & Green, 2009), even in the most conflictual intergroup contexts (Al Ramiah & Hewstone, 2013) and even in the face of discrimination and threat perceptions (Van Assche et al., 2023). Hence, interventions conducted using the contact paradigm are indicated to be highly effective in combating prejudice and discrimination within school settings (e.g. Beelmann & Heinemann, 2014; Bigler & Liben, 2007; Tropp et al., 2022; Turner & Cameron, 2016).

In educational contexts where the ratio of majority vs. minority group students may be unbalanced, direct contact interventions may be theoretically possible but still carry potential impracticalities (see also Di Bernardo et al., 2021). These findings suggest that, particularly when face‐to‐face contact is difficult or involves potential risks, indirect forms of contact may stand as effective tools and improve intergroup attitudes and behaviours successfully in naturalistic environments such as schools (e.g. White et al., 2020). While there is no universally accepted definition of the term in the contact literature, *indirect contact* has been largely used as an umbrella term to define various forms of contact experiences that are, in theory and practice, not *direct*, therefore not requiring or including a face‐to‐face interaction (see White et al., 2021, for a recent review). Whereas indirect contact referred only to *extended contact*, which occurs through observing or witnessing ingroup members' contact with the outgroup in earlier research (e.g. Pettigrew et al., 2008; Wright et al., 1997), later studies indicated a broader approach and showed indirect contact to occur through different means and processes, such as mass media, computers or mental stimulation. Indirect contact could be formed through knowing that an ingroup member has a positive relationship with an outgroup member (extended contact), imagining a positive interaction with an outgroup member (imagined contact), observing such an interaction (vicarious contact) or interacting with an outgroup through mass media or computers (mass‐mediated contact and e‐contact). For example, vicarious contact through storytelling and video watching (e.g. Cocco et al., 2021; Husnu et al., 2018; Liebkind et al., 2014) and repeated applications of imagined contact interventions (Vezzali et al., 2012), which have been thoughtfully crafted for classroom use and children's developmental stages, have been found to improve children's attitudes towards diverse groups such as immigrant, ethnic minority and disabled children (see also Di Bernardo et al., 2021).

However, even the typically established indirect contact interventions may result in complex social psychological processes that restrict the formation of positive relationships (e.g. Brown & Paterson, 2016). For example, previous research testing imagined contact—an indirect contact procedure where participants are asked to simply imagine an intergroup contact (Turner et al., 2007)—has revealed that although participants were instructed to imagine a positive contact, they may still imagine negative experiences of contact (Bagci et al., 2018) or perceive imagining experience as difficult (Birtel & Crisp, 2012). While vicarious contact experiments are particularly promising, demonstrating both storytelling and video watching as equally effective strategies (Cocco et al., 2021), such interventions may not easily generalize to hostile intergroup contexts. As such, testing a vicarious contact intervention among local Turkish children, Tercan et al. (2021) found that the intervention was effective in increasing helping intentions, only among children with higher initial prejudice. While this finding highlights the value of existing strategies, it also points to the need for novel approaches that operate through different mechanisms. Specifically, interventions that move beyond passive reception and actively involve children as engaged participants in fun, positive experiences may offer a complementary potential for improving intergroup dynamics. In the current studies, we tested the effectiveness of a novel contact procedure where native children formed an indirect interaction with their Syrian peers through a gift exchange process.

### Reciprocal gifting as a potential contact intervention

In the current study, we tested contact through reciprocal gift sharing, which is conceptualized as the exchange of objects with shared symbolic value, serving as the foundation of transactions between human groups or group members and facilitating improvements in intergroup relations (Mauss, 1967). According to Mauss, reciprocal gifting is one of the most fundamental ways in archaic societies to demonstrate goodwill and a positive intent for building contact with another group. The offering and receiving of objects with shared value, according to Mauss, facilitate bond formation between communities and provide a significant opportunity for communities to recognize one another. Gifts often have symbolic associations that imply an invitation to the partner and could be seen as an expression of a social relationship (Sherry, 1983), and the following mutual feeling of gratitude has been suggested to be a strong motivator of social cohesion (Komter, 1996).

#### Supportive studies on reciprocal gifting and prosocial exchange

Prosocial behaviours in intergroup contexts have long been discussed as effective tools to reduce prejudice and restore trust. Acts such as helping, apologizing, cooperation and gift exchange across different groups signal benevolent intent to the outgroup and can elicit positive affect (Çoksan & Çakmak, 2025; Reinders Folmer et al., 2021; Taylor et al., 2014). Neuroscientific evidence further suggests that gift sharing may increase interpersonal attunement and coordination (Balconi & Fronda, 2020). Reciprocal gifting is a tangible prosocial act involving elements such as intention and resource transfer. In this context, the reciprocal gifting process may allow participants to position themselves as recipients of the prosocial intentions of outgroup members and then reciprocate this intention with their own prosocial action (Dovidio et al., 2006), and such reciprocity may contribute to the development of a shared prosocial identity.

#### Theoretical justification of reciprocal gifting as indirect intergroup contact

In the current research, we conceptualized reciprocal gifting as a potential indirect contact strategy for several reasons. Direct contact, in its most fundamental definition, refers to face‐to‐face encounters between members of different groups that occur in a shared space and typically allow for synchronous interaction or immediate feedback; such encounters enable individuals to acquire personal and category‐defying information about one another, thereby weakening stereotypes (Allport, 1954). Indirect contact, by contrast, is defined by the absence of a face‐to‐face encounter and exerts its influence through alternative psychological pathways such as mental simulation, social observation or social networks (Turner et al., 2007; Vezzali & Stathi, 2017). While previous research suggested meeting with ostensible outgroup partners and anticipated contact procedures to function as direct contact (e.g. Vorauer & Sasaki, 2009), our procedure did not include the possibility of copresence, synchronicity and/or immediate feedback and could therefore be described as an *object‐mediated indirect contact*. Hence, reciprocal gifting can be conceptualized as an *object‐mediated* and *asynchronous* form of *indirect intergroup exchange*. This rationale can be evaluated through Harwood's (2010) *contact space* framework. Our procedure entails relatively high self‐involvement because children personally receive and open a gift they believe came from an outgroup peer and then, under an obligation to reciprocate, prepare and send a return gift. This self‐involvement is further amplified in Study 2, as children produce a symbolic and personalized return gift (for example, drawings and messages on a marble canvas), thereby incorporating agency, effort and self‐expression more strongly into the process. By contrast, the richness of the self‐outgroup experience is constrained because the procedure lacks copresence, synchronicity and immediate feedback. The outgroup is represented through limited cues, such as a photograph and a voice recording, and through a tangible object (in this paper, the gift), so the interaction is experienced as an indirect exchange mediated by objects rather than as a face‐to‐face encounter. Building on Harwood's (2021) discussion of modes of intergroup contact, this procedure can be viewed as a bounded contact mode with high practical applicability in settings where direct contact is discouraged by hostility or by prevailing norms. The procedure may elicit spontaneous mental simulation for some participants; however, because children are not instructed to do so, it may not be classified as one of the established indirect contact paradigms in the literature (e.g. imagined contact). The theoretical novelty of our procedure may lie in our proposal of a unique indirect contact taxonomy that occurs through *symbolic reciprocal exchange*. This approach offers a theoretical innovation by bridging the contact and prosociality literatures, while also functioning as a practical strategy in highly prejudicial contexts where direct contact may not be practically feasible or safe.

#### Potential psychological processes underlying reciprocal gifting

Several other mechanisms may be suggested to argue that reciprocal gifting is likely to constitute a form of indirect intergroup contact whereby intergroup relationships may be improved. We based this assumption on a few key attributes of the reciprocal gifting procedure. First of all, reciprocal gifting, as an indicator of friendship and mutual respect, is likely to involve a level of interactional intimacy and the engagement of the self, where the participants actively create a social bond with the receivers, providing a positive and intimate social situation that is key in the effectiveness of contact strategies on improving attitudes (e.g. Marinucci et al., 2021).

Second, the procedure capitalizes *reciprocity*, which signals positive contact meta‐perceptions (i.e. the perception that the outgroup member is willing to engage in positive contact), often suggested as one of the boosters of indirect contact strategies (Matera et al., 2024). While traditionally studied within dyadic relationships, recent theoretical frameworks have extended reciprocity's relevance to intergroup contexts, suggesting its critical role in shaping intergroup attitudes and behaviours (Perugini et al., 2003). In intergroup settings, reciprocity is not merely transactional but is instead a socially regulated process shaped by identity dynamics, perceived intentionality and internalized moral obligations, which may in turn provide an ideal contact context for the development of positive intergroup relationships.

Third, at an affective level, the gifting process often involves positive emotions such as joy, pride and gratitude in return (e.g. Ruth, 1996). Gift sharing is also likely to involve the excitement and fun aspect of intergroup contact experiences, which is often overlooked in tightly structured contact interventions (see Amichai‐Hamburger et al., 2015; Amichai‐Hamburger & McKenna, 2006). Hence, especially in a prejudicial context, a positive and fun experience involving the outgroup might provide critical cues about the positive intentions of the outgroup, which may eventually create more positive perceptions about the outgroup and decrease prejudicial attitudes. Therefore, we anticipated that the intervention might generate effects similar to those observed in the general indirect contact literature (e.g. Gómez & Huici, 2008; Mazziotta et al., 2011; Vezzali et al., 2014; White et al., 2021).

### The current study

Through two experimental studies, we tested whether reciprocal gifting was effective in shaping the attitudes of local (Turkish) children towards their Syrian refugee peers, assessed by both positive and negative attitudes and social distance (closeness) towards the outgroup. We treated positive and negative attitudes separately, since these have been found to be conceptually and empirically distinct (e.g. Cacioppo & Berntson, 1994) and in the context of intergroup contact, gifting may primarily activate pathways such as positive affect, gratitude, agency and a sense of normative closeness, and by contrast, negative attitudes may be more structural and therefore more resistant to change (Gim & Harwood, 2024, 2025). We further used social distance as a behavioural indicator of attitudes, given that previous research has demonstrated contact to have differential outcomes in terms of attitudes and social distance (Bastian et al., 2012). Conceptual and empirical justifications for the distinction between these constructs were provided in Data S1. In both studies, we used a 2 by 3 mixed‐design whereby children in control and experimental conditions were tested before the reciprocal gifting procedure (pre‐test), immediately after (post‐test) and approximately 40 days later (follow‐up). In Study 1, children in the experimental condition received gifts that were told to come from their Syrian peers and send similar gifts to them, whereas in Study 2, the procedure involved more engagement during the gift preparation. For ethical considerations, the reciprocal gifting procedure was also implemented among students in the control group after the follow‐up (see Data S2). We expected that the procedure would increase positive outgroup attitudes (*H*_*1a*_), decrease negative outgroup attitudes (*H*_*1b*_) and enhance social closeness (*H*_*1c*_) among children in the experimental condition. Moreover, immediately after the intervention, we hypothesized that the experimental group would have a greater positive attitude (*H*_*1d*_), a less negative attitude (*H*_*1e*_) and social closeness (*H*_*1f*_) compared with the control group. Additionally, we hypothesized that these effects would persist during follow‐up measurements (H_2a‐c_).

The current study contributes to contemporary intergroup relations literature in several ways. First of all, to our knowledge, reciprocal gifting has not been tested previously as a strategy to improve attitudes in an intergroup context, nor has it been suggested as a form of indirect contact and practically applied among children. We believe reciprocal gifting may provide additional benefits to the typically used indirect contact strategies by demonstrating a concrete prosocial action from the outgroup, followed by reciprocation; involving the engagement of the self in the process; and stimulating a fun and pleasant experience for children. Second, given that direct contact is difficult to implement in highly prejudicial intergroup contexts, reciprocal gifting may function as an effective tool to decrease the existing tensions between the two groups, which is known to particularly exist in school environments with Syrian refugee children (Bagci et al., 2023). Recently, scholars have suggested that indirect contact strategies are not simple *alternatives* to direct contact, but they have their unique benefits over and beyond direct contact (White et al., 2021). Third, unlike the majority of indirect contact experiments that involve the assessment of outcome variables only at a single time point (e.g. Cocco et al., 2021; Vezzali et al., 2019, but see Vezzali et al., 2015), we also assessed the stability of effects, which provides a fuller picture of how the strategy shifted attitudes over time.

## STUDY 1

Study 1 tested the effectiveness of the reciprocal gifting procedure on Turkish children's attitudes and closeness to their Syrian refugee peers.

### Method

The dataset, codebook, visual stimulus, measurement tools and supplementary referenced in the text are available at the following OSF link: https://osf.io/768gt/?view_only=5bffa58efe74487490c15786f5ef11da. Sensitivity analyses for significant results of Study 1 and Study 2 are reported in Data S3.

#### Participants and procedure

We randomly selected eight out of 21 classrooms in a state primary school located in İzmir, Türkiye. Among the 236 students enrolled in these classrooms, 30 were refugees, and the percentage of refugees across classrooms ranged between approximately 6% and 22%. Since the manipulation specifically targeted the majority group members, Syrian refugee students were not included in the data collection and engaged in a non‐relevant task. Out of 206 Turkish students, the parents of five students did not grant permission for participation, three students chose not to participate, 11 students changed schools during the experimental period and 43 students missed at least one of the three experimental sessions. The final sample included 144 students (76 female, 68 male; *N*_experimental_ = 88, *N*_control_ = 56), with a mean age of 9.49 years (SD = .52; age range: 9–11 years).

After obtaining ethics committee approval numbered 10/3‐27.10.2022 from the Erzurum Technical University Ethics Committee and the procedural approval from the Turkish Republic Ministry of Education, we presented the project details to the education board of the school where the study would be conducted. We provided parental consent forms to the classroom teachers in the selected classrooms, asking them to distribute the forms to parents and collect them afterward. Before the first measurement, we explained the implicit purpose of the study to the students and asked them to provide verbal consent for participation. Students whose parents did not provide consent, as well as those who expressed their unwillingness to participate, were taken to a separate classroom and engaged in an interactive game with the teacher, which was repeated at all measurement sessions (pre‐test, post‐test, follow‐up).

The participating children received booklets containing the main measures, accompanied by two research assistants, an assistant principal, a teaching assistant and the respective classroom teacher. We conducted the pre‐test measurements within the same week, starting from the third Monday of February 2023, and the entire process took approximately 40 minutes per classroom. Approximately 45 days later, we revisited the school and, after obtaining verbal consent from students randomly assigned to the experimental group, implemented the reciprocal gifting procedure. The control group engaged in a 40‐min interactive story‐reading activity that was matched in duration to the gifting procedure. The story, which had a thematic focus on sharing and prosocial behaviour among animals (e.g. rabbits sharing carrots), was unrelated to intergroup relations. A researcher read the story aloud, pausing at predetermined points to ask students standardized questions about their feelings and opinions (e.g. How would you feel if you were in this situation?). At the end, students were invited to suggest how the story might conclude. We then collected post‐test measurements from all participants, and this phase took approximately 80 min per classroom. After another 45 days, we returned to the school to collect follow‐up measurements from the students (approximately 40 min). To ensure that participants in the control condition were not deprived of the potential positive effects of the reciprocal gifting procedure, we revisited the school about a week later and implemented the gifting procedure for the control group participants after the follow‐up session. Finally, all students and parents were debriefed about the main purpose of the study and were allowed to keep the gifts they received during the gifting procedure. Across all sessions, the classroom teacher and the research team were present in all conditions, and at each session, we reiterated that participation was voluntary and that non‐participation would have no academic or social consequences.

#### The pilot study

For the pilot study, two separate colour portrait images depicting a typical Syrian male child and a typical Syrian female child were generated using artificial intelligence (AI) tools. The images were designed to be age‐appropriate (approximately 9–10 years old) for the participants in the main study, against a plain background and in everyday clothing. The full set of visual stimuli is available in the OSF repository (see Data S4). To test whether students would perceive these stimuli as real, we conducted a pilot study. A total of 46 volunteers (80.4% female, 19.6% male) with a mean age of 20.3 years (SD = 1.65) participated in this pilot study and attempted to determine the social identity (Syrian vs. Turk) of two AI‐generated images of Syrian children. For the Syrian male child image, 40 (87%) participants stated that the image was of a Syrian; for the Syrian female child image, 34 (74%) participants stated that the image was of a Syrian. A chi‐square analysis indicated that participants were significantly more likely to classify the images as belonging to Syrian individuals, both for the image of a male child (*p* < .001) and a female child (*p* < .001).

#### The reciprocal gifting manipulation

Children in the experimental group were initially (before the post‐test) informed that two Syrian refugee children had organized their own classrooms to send them gifts. We also projected images of these (hypothetical) Syrian refugee children onto the smartboards in the classrooms and played an audio message through the speakers, informing the students that these children had sent them gifts. Following this, a researcher distributed pre‐prepared gift packages wrapped in coloured paper to the students. Each package contained one pencil, one eraser, one sharpener, a set of 12 coloured pencils and a notebook. After opening their gifts, the students listened to a thank‐you voice recording, which we told them had been recorded by their Syrian refugee peers. Next, we distributed similar gift items, wrapping papers in various colours and tape, instructing the students to prepare their own gift packages. We informed them that they could include personal thank you messages in their packages if they wished. Finally, we collected all the gift packages, telling the students that they would be sent to the Syrian refugees who had originally sent them gifts.

#### Measures

We asked participants to report their age and gender in the demographic information form. Additionally, to provide a more detailed descriptive analysis, we asked them in the pre‐test how many immigrant friends they had (*How many immigrant friends do you have who are around your age*?) and how satisfied they were with their friendships with these friends (*If you have any immigrant friends, how satisfied are you with your friendship with them?*). To ensure clarity for the child participants, both items were accompanied by the following definition and example: *Immigrants are people who were born outside of Türkiye but currently live in Türkiye. For example, your Syrian friends are immigrants*. Given this explicit prompt, participants' responses predominantly reflected their relationships with their Syrian peers. The average number of friends reported by participants was 3.83 (SD = 3.02), and their satisfaction score with these friendships (2.66 out of 3, SD = .59) was also assessed. We also asked participants at the end of the experiment how much they liked the gifts they received from their Syrian peers (‘How much did you like the gifts sent to you by your Syrian peers?’). Their satisfaction with the gifts presented in the experiment (3.81 out of 4, SD = .44) was high.

We used a 14‐item adjective list (Çoksan, 2016) to measure participants' *positive and negative attitudes* towards outgroup members. Participants evaluated outgroup members using this list, which consisted of seven positive adjectives (e.g. kind, helpful; α_pre‐test_ = .86, α_post‐test_ = .88, α_follow‐up_ = .92) and seven negative adjectives (e.g. rude, selfish; α_pre‐test_ = .84, α_post‐test_ = .86, α_follow‐up_ = .92) on a 5‐point Likert scale (0 = not at all, 4 = absolutely). We conceptualized the mean score of positive attribute ratings obtained from this measure as representing positive attitudes towards the outgroup and the mean score of negative attribute ratings as representing negative attitudes towards the outgroup (see Data S1).

We adapted Bogardus's (1925) Social Distance Scale to the research context to measure participants' *social closeness* towards their Syrian friends (see Bagci et al., 2022). The 10‐item scale, structured as a 5‐point Likert‐type measure (0 = No, I would not be happy at all, 4 = Yes, I would be very happy), begins with participants evaluating their parents working at the same workplace as an outgroup member's parents and progresses to assessing how happy they would be about the possibility of marrying an outgroup member in the future (α_pre‐test_ = .87, α_post‐test_ = .90, α_follow‐up_ = .92). The higher scores on this scale indicate greater closeness (i.e. lower social distance) to outgroup members.

### Results and discussion

A 2 by 3 mixed‐design repeated‐measures ANOVA^1^ was conducted to examine the effect of time (pre‐test, post‐test, follow‐up) and group (experimental, control) on positive attitudes.^2^ The interaction effect between time and group was significant (*F*(2,284) = 7.11, *p* < .001, η^2^ = .016), suggesting that changes in positive attitudes over time differed across groups. Post‐hoc pairwise comparisons demonstrated noticeable improvement in the experimental group, with positive attitudes significantly increasing from the pre‐test (*M* = 2.61, SE = .09) to the post‐test (*M* = 2.85, SE = .09; *t*(142) = −2.27, *p* = .025), but decreasing significantly from the post‐test to the follow‐up (*M* = 2.40, SE = .11; *t*(142) = 2.34, *p* = .021). There was no difference between pre‐test and follow‐up (*t*(142) = 1.93, *p* = .055). The experimental group also displayed more positive attitudes than the control group, particularly at the post‐test (*M* = 2.41, SE = .11; *t*(142) = 2.16, *p* = .032). For the control group, there were no significant changes across the three time points.

We ran another mixed‐design repeated‐measures ANOVA for the outcome of negative attitudes. The interaction effect between time and group type was not significant (*F*(2,284) = 0.94, *p* = .394, η^2^ = .002), indicating that changes in negative attitudes over time did not differ significantly between the two groups. The results of another mixed‐design repeated‐measures ANOVA for social closeness showed that the interaction between time and group type was significant (*F*(2,284) = 4.70, *p* = .010, η^2^ = .008). Post‐hoc pairwise comparisons demonstrated that social closeness significantly decreased from the pre‐test (*M* = 3.38, SE = .09) to the follow‐up (*M* = 2.81, SE = 0.11; *t*(142) = 4.84, *p* < .001). However, there was no difference between the pre‐test and the post‐test (*M* = 3.22, SE = .10). Moreover, the experimental group had a higher social closeness than the control group at the post‐test (*M* = 2.80, SE = 0.13; *t*(142) = 2.61, *p* = .010). For the control group, social closeness ratings also significantly decreased from the pre‐test (*M* = 3.41, SE = 0.12) to the post‐test (*t*(142) = 5.40, *p* < .001) and from the post‐test to the follow‐up test (*M* = 2.55, SE = 0.14, *t*(142) = 2.25, *p* = .026), demonstrating a steady decline in social closeness over time. Initially, all models were run as ANCOVAs, including the number of Syrian friends, satisfaction with these friendships and satisfaction with the received gifts as covariates. However, since none of these covariates showed a significant effect in any of the models, we followed the common practice and reported the simpler and more powerful ANOVA models.

**FIGURE 1:**
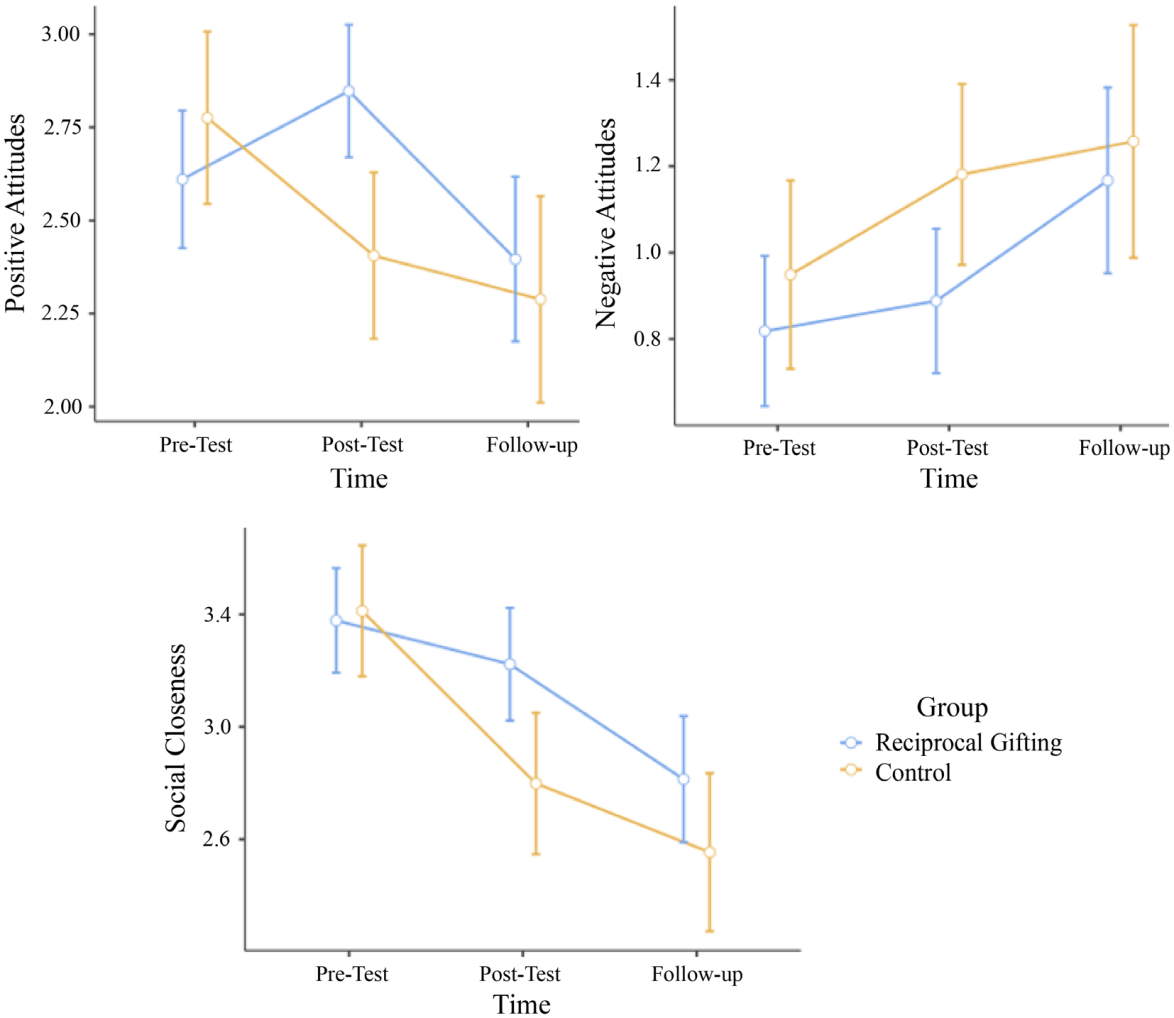
Changes in positive attitudes, negative attitudes and social closeness across time by condition in Study 1.

In summary (see Figure 1), findings of Study 1 demonstrated that the reciprocal gifting procedure resulted in *short‐term* effects on positive attitudes (*H*_*1a*_), which disappeared during the follow‐up, contrary to our hypothesis (*H*_*2a*_). We also found no significant effects on negative attitudes (*H*_*1b*_). Nevertheless, these findings provide partial evidence for our initial expectations (*H*_*1d*_, *H*_*1e*_, & *H*_*1f*_) and point to the potential role of reciprocal gifting in improving attitudes, demonstrated by greater positivity observed in the experimental group compared with the control group during post‐tests. However, the procedure seemed to mostly *buffer* the growing negativity in attitudes and behaviours over the school semester, which is a finding that has been observed in a similar Turkish‐Syrian refugee school setting (see Bagci et al., 2023). The lack of significant effects, particularly on negative attitudes, also points to the persistence of negative attitudes over time, overall requiring the testing of a more enhanced procedure.

## STUDY 2

We designed pre‐registered Study 2 (https://osf.io/768gt/?view_only=5bffa58efe74487490c15786f5ef11da) in response to several limitations identified in Study 1. The reciprocal gifting procedure led to short‐lived improvements in positive attitudes and social closeness, with these effects fading by the follow‐up period. We speculated that participants' relatively low motivation and engagement, though not directly measured, might have limited the effectiveness of the intervention. This assumption was based on classroom observations and informal feedback from teachers, suggesting that students may not have perceived the activity as personally meaningful or effortful. In Study 1, children simply repackaged items handed by researchers, without engaging in creative effort or expressing personal agency. This lack of symbolic personalization and volitional investment might have diminished the moral significance of the exchange and weakened the emergence of a sense of obligation or mutual recognition (e.g. Üzümçeker & Akfırat, 2024). It is possible, therefore, that the limited behavioural involvement required by the procedure may not have capitalized on the critical elements in the process.

Study 2 aimed to test whether a more comprehensive and contextually grounded version of the reciprocal gifting procedure could generate stronger and more sustained improvements in intergroup attitudes, compared with two conditions—the simpler gifting procedure (the original reciprocal gifting) and the control conditions (no reciprocal gifting) that were assessed in Study 1. In particular, we make some of the potential components, such as reciprocity, engagement of the self and symbolic meaning, more salient to increase the overall effectiveness of the procedure. In this version, children were provided with small marble canvases and painted materials and were invited to design personalized artworks that were subsequently sent as reciprocal gifts for the same outgroup members. This revised approach aligned more closely with theoretical accounts of intergroup reciprocity by emphasizing personal authorship, expressive content and meaningful engagement. In this study, we referred to this procedure as the *enhanced reciprocal gifting*. By fostering greater psychological investment in the act of giving, this version of the intervention was expected to promote stronger and more enduring changes in intergroup attitudes based on mutual acknowledgment and moral connection.

### Method

#### Participants and procedure

Data were collected from 16 classrooms randomly selected from the same school (and grade with a new cohort) as in Study 1. Among the 319 students enrolled in these classrooms, 33 were refugees. As in Study 1, only Turkish students were included in the data collection. There were no students whose parents withheld consent or who personally declined to participate. An attention check item (i.e. ‘Please select *Strongly disagree* for this item’) was embedded within the measurement tools in the data collection booklet. Thirty‐nine students failed this check during the pre‐test phase, 22 during the post‐test phase and nine during the follow‐up phase. Additionally, nine students missed at least one of the three data collection sessions, resulting in a final sample size of 207 students (*N*_ReciprocalGifting_ = 48, *N*_EnhancedReciprocalGifting_ = 79, *N*_Control_ = 80). The mean age of the participants was 9.50 years (SD = .55; age range: 8–11 years), with 118 females and 89 males. The average number of Syrian friends (outgroup members) reported by the participants was 3.83 (SD = 3.02). The mean number of Syrian friends was 3.39 (SD = 2.78), and the average satisfaction score with these friends was 3.60 out of 5 (SD = 1.12).

After obtaining the ethical approvals, during the pre‐test, children were presented with a booklet containing the overall measures and took approximately 40 minutes per classroom (completed in the first week of March 2024). Children were randomly assigned to three conditions; the less elaborated reciprocal gifting group engaged in the exact same task as in Study 1, where we implemented the same gifting procedure used in Study 1; the more elaborated, enhanced reciprocal gifting condition introduced in Study 2; and the control group participated in a 90‐min interactive story‐reading session, matched in duration to the enhanced gifting procedure. The activity used a story with a prosocial theme unrelated to intergroup contexts. The session involved the researcher reading the story, asking predetermined interactive questions to engage students' perspectives and allowing students to collaboratively determine the story's ending. Following this, we collected post‐test data from all participants, with the entire process lasting approximately 120 min per classroom. About 30 days later, we returned to the school to collect follow‐up data (30 min). To ensure that the control group was not deprived of the potential positive effects of the gifting procedure, we revisited the school about a week later and implemented the new gifting procedure for them as well (see Data S6). Finally, we debriefed all students and parents, and all stationery items received during the gifting procedure were given to the children as incentives.

#### The enhanced reciprocal gifting manipulation

In this new enhanced reciprocal gifting procedure, the process of introducing the two Syrian refugee children and receiving the gifts was identical to the process implemented in Study 1. After being given the gifts (hypothetically coming from the Syrian peers), children were provided with small marble canvases. They were instructed to use colouring materials to draw images expressing their feelings and were told they could also write positive emotions and wishes on the canvases if they liked. After completing their drawings, the students sealed and kiln‐fired the marble pieces. Once finalized, the marble canvases were wrapped in coloured papers and taped into gift packages. Students were also allowed to include their personal thank you messages in the packages. When collecting the packages, we told the participants that these would be sent to the Syrian refugee children who had originally sent them gifts, thus making reciprocity and engagement salient during the procedure.

#### Measures

To measure participants' attitudes towards outgroup members, we used the same adjective list from the previous study, presented on a 5‐point Likert scale (1 = Not at all, 5 = Absolutely) (for the positive adjective list, α_pre‐test_ = .86, α_post‐test_ = .87, α_follow‐up_ = .90; for the negative adjective list, α_pre‐test_ = .85, α_post‐test_ = .91, α_follow‐up_ = .90). In the previous study, the last two items of the social distance scale involved potential future romantic relationships. Many students across classrooms expressed discomfort when responding to these items. Therefore, in Study 2, we used a modified version of the same scale, omitting the last two items. This resulted in an 8‐item version administered on a 5‐point Likert scale (1 = No, I would not be happy at all, 5 = Yes, I would be very happy) (α_pre‐test_ = .90, α_post‐test_ = .91, α_follow‐up_ = .93).

### Results and discussion

We conducted a 3 by 3 mixed‐design repeated‐measures ANOVA^3^ to examine the effect of time (pre‐test, post‐test, follow‐up) and group (reciprocal gifting condition, enhanced reciprocal gifting condition and control group) on positive attitudes. The interaction effect between time and group was significant (*F*(4,408) = 6.02, *p* < .001, η^2^_p_ = .056), suggesting that changes in positive attitudes over time differed based on the group type. Post‐hoc pairwise comparisons showed that there was no difference between the reciprocal gifting condition (*M* = 3.17, SE = .10), the enhanced reciprocal gifting condition (*M* = 3.27, SE = .12) and the control condition (*M* = 3.41, SE = .10) in terms of their pre‐test scores. Positive attitudes significantly increased from the pre‐test to the post‐test (*M* = 3.59, SE = .09; *t*(204) = −4.71, *p* < .001) but decreased significantly from the post‐test to the follow‐up test (*M* = 3.19, SE = .11; *t*(204) = 4.70, *p* < .001) for the reciprocal gifting condition. The same pattern was observed for the enhanced reciprocal gifting condition. Positive attitudes significantly increased from the pre‐test to the post‐test (*M* = 3.83, SE = .12; *t*(204) = −4.86, *p* < .001) but decreased significantly from the post‐test to the follow‐up test (*M* = 3.26, SE = .14; *t*(204) = 5.13, *p* < .001). For the control group, there was no significant difference between pre‐test and post‐test (*M* = 3.35, SE = .09), and also between post‐test and follow‐up (*M* = 3.19, SE = .11). However, positive attitudes were higher at pre‐test than at follow‐up (*t*(204) = 2.40, *p* = .017) in the control group.

**FIGURE 2:**
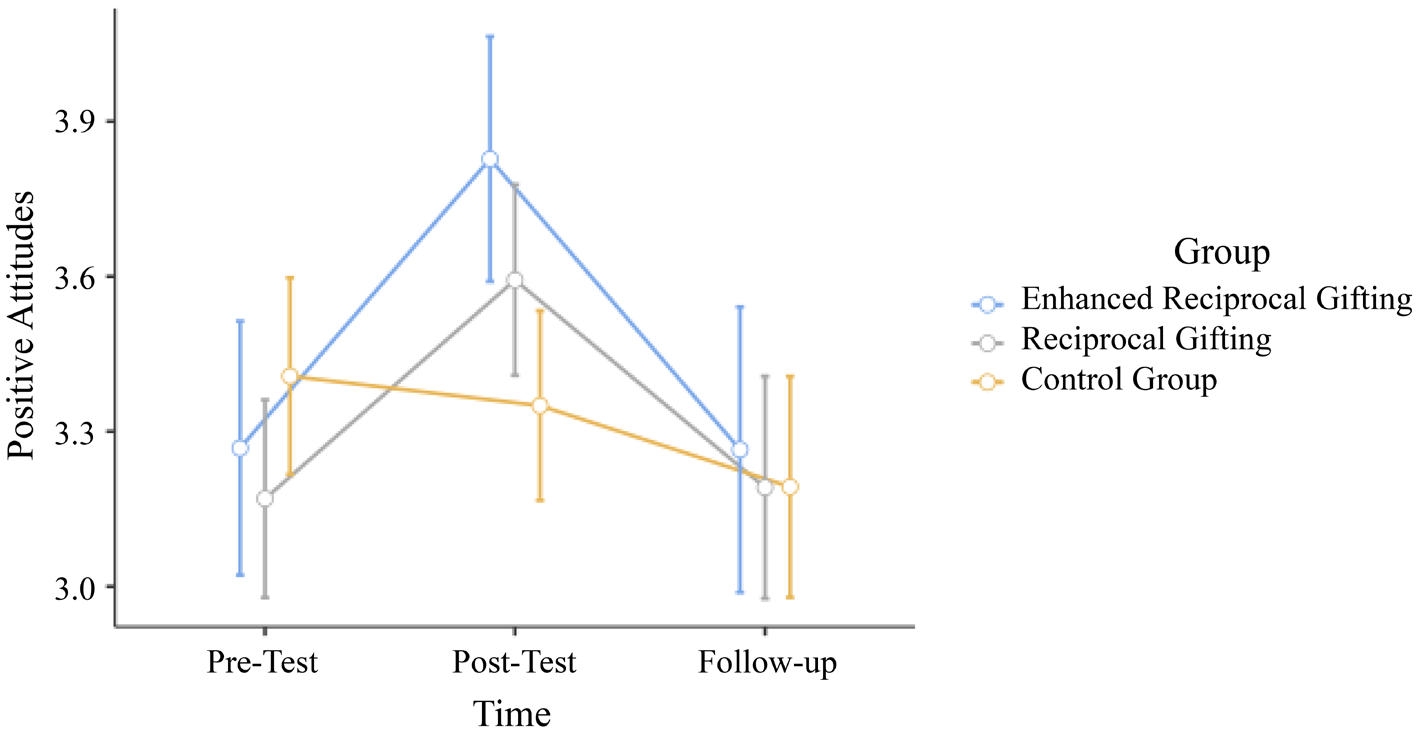
Changes in positive attitudes towards Syrian refugee peers across three conditions at pre‐test, post‐test and follow‐up.

Although there was no difference between the enhanced reciprocal gifting condition and the reciprocal gifting condition for the post‐test scores, only the new reciprocal gifting condition resulted in improved positive attitudes compared with the control condition during the post‐test (*t*(204) = 3.14, *p* = .002). In terms of the follow‐up measurements, as in the pre‐test, there were no significant differences among the three conditions. Results regarding positive attitudes are summarized in Figure 2 below.

We ran another mixed‐design repeated‐measures ANOVA for negative outgroup attitudes, and as in Study 1, the interaction effect between time and group was not significant (*F*(4,408) = 1.85, *p* = .112, η^2^_p_ = .018), indicating that changes in negative outgroup ratings did not differ significantly between the two groups. Post‐hoc comparisons also showed no significant difference between reciprocal gifting and enhanced reciprocal gifting conditions, and enhanced reciprocal gifting and the control group in terms of negative attitudes on the post‐test measurements.

**FIGURE 3:**
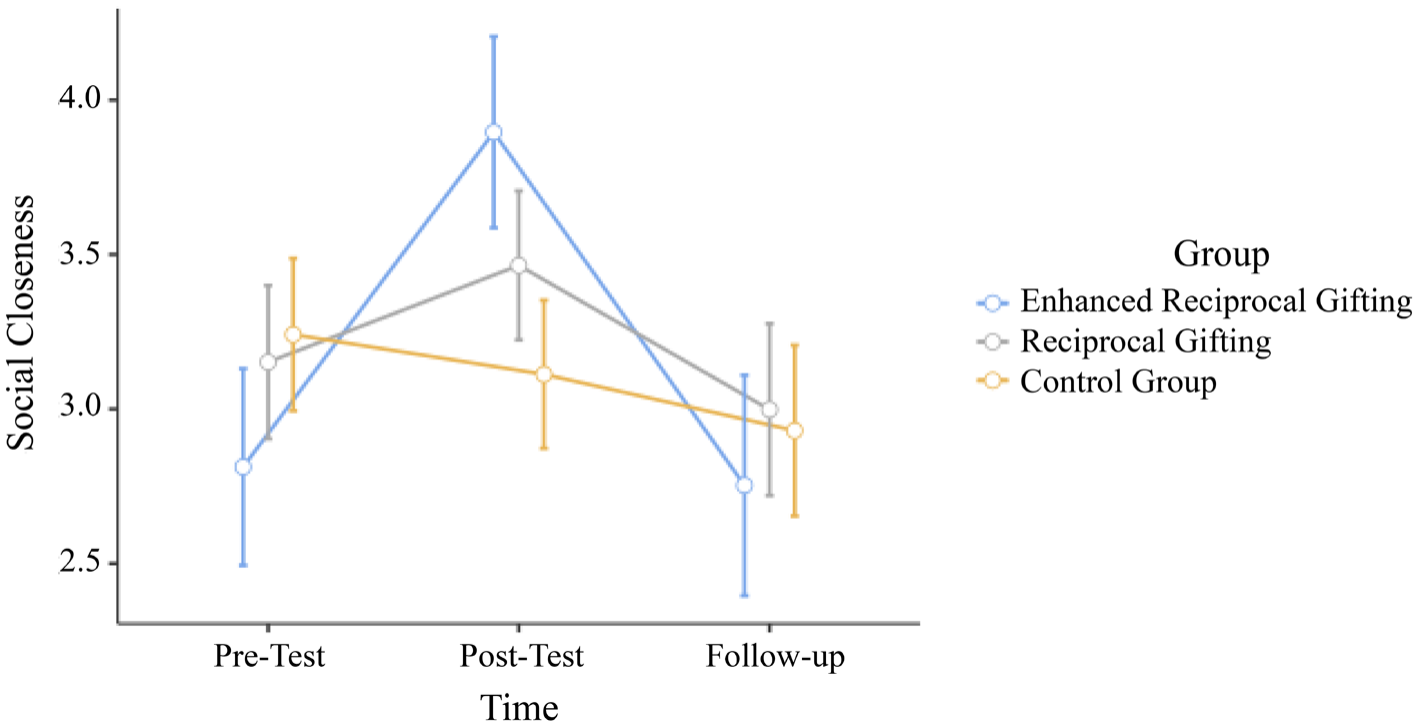
Changes in social closeness towards Syrian refugee peers across three conditions at pre‐test, post‐test and follow‐up.

The results of another mixed‐design repeated‐measures ANOVA for social closeness outcome showed that the interaction effect between time and group type was significant (*F*(4,408) = 13.90, *p* < .001, η^2^_p_ = .120). Post‐hoc pairwise comparisons showed that participants in the enhanced reciprocal gifting condition had significantly higher post‐test scores (*M* = 3.90, SE = .16) compared with their pre‐test scores (*M* = 2.81, SE = .16; *t*(204) = −8.05, *p* < .001), but their scores declined to the baseline level at the follow‐up (*M* = 2.75, SE = .18; *t*(204) = 8.00, *p* < .001), indicating that follow‐up scores returned to pre‐test levels (*t*(204) = .46, *p* = .649). A similar pattern was observed for the reciprocal gifting procedure developed in Study 1, with participants in the reciprocal gifting condition reporting higher social closeness towards their Syrian peers at the post‐test (*M* = 3.46, SE = .12) compared with the pre‐test (*M* = 3.15, SE = .12; *t*(204) = −2.98, *p* = .003). However, follow‐up measurements (*M* = 3.00, SE = .14) revealed a reversal of this trend, showing that social closeness returned to pre‐test levels (*t*(204) = 1.51, *p* = .113). On the contrary, participants in the enhanced gifting condition had significantly higher post‐test scores (*M* = 3.90, SE = .16) than those in the original gifting condition (*t*(204) = 2.17, *p* = .031) and the control group (*M* = 3.11, SE = .12; *t*(204) = 3.95, *p* < .001). Results regarding social closeness are summarized in Figure 3. As in Study 1, the number of Syrian refugee friends, the average satisfaction with these friendships and the average satisfaction with the received gifts did not show any significant covariate effects in any of the models; hence, we reported the simpler and more powerful ANOVA models.

When considered collectively, the results of Study 2 suggest that the enhanced reciprocal gifting procedure produced short‐term improvements in positive attitudes (*H*_*1a*_ & *H*_*1d*_) and social closeness (*H*_*1c*_ & *H*_*1f*_), and yielded stronger social closeness compared with the original version; however, consistent with Study 1, these effects did not persist over time (*H*_*2a*_ & *H*_*2c*_). These findings imply that although enhancing participant agency and symbolic engagement can amplify the immediate impact of intergroup interventions, sustaining these effects over time remains a significant challenge.

We are aware that contact interventions may be particularly effective among members holding higher initial prejudice (see Hodson et al., 2016). Therefore, for the models we examined in both Study 1 and Study 2, we also investigated whether participants' pre‐test attitude measures served as a moderator; however, since we found that none of the initial attitudes played a moderating role, we have reported these analyses in Data S7. The correlation between outcomes and their descriptives in both studies is presented in Table 1 below.

**TABLE 1:** The table of correlations between outcomes and their descriptives.

	(I)	(II)	(III)	(IV)	(V)	(VI)	(VII)	(VIII)	(IX)
Pre‐test positive attitudes (I)		−.71***	.61***	.52***	−.51***	.37***	.62***	−.50***	.45***
Pre‐test negative attitudes (II)	−.65***		−.53***	−.43***	.64***	−.34***	−.51***	.60***	−.39***
Pre‐test social closeness (III)	.51***	−.33***		.38***	−.39***	.58***	.52***	−.39***	.71***
Post‐test positive attitudes (IV)	.32***	−.21*	.25**		−.64***	.60***	.64***	−.53***	.43***
Post‐test negative attitudes (V)	−.41***	.39***	−.32***	−.45***		−.42***	−.64***	.74***	−.41***
Post‐test social closeness (VI)	.39***	−.25**	.56***	.47***	−.53***		.48***	−.43***	.61***
Follow‐up positive attitudes (VII)	.42***	−.32***	.35***	.66***	−.59***	.53***		−.78***	.73***
Follow‐up negative attitudes (VIII)	−.29***	.39***	−.19*	−.38***	.63***	−32***	−.71***		−.62***
Follow‐up social closeness (IX)	.35***	−.26**	.50***	.45***	−.46***	.68***	.64***	−.50***	
	*M* = 2.68 SD = .88 *M* = 3.28 SD = .87	*M* = 0.87 SD = .83 *M* = 2.09 SD = .82	*M* = 3.39 SD = .88 *M* = 3.11 SD = 1.13	*M* = 2.68 SD = .87 *M* = 3.55 SD = .85	*M* = 1.00 SD = .80 *M* = 2.08 SD = .93	*M* = 3.06 SD = .97 *M* = 3.43 SD = 1.12	*M* = 2.35 SD = 1.05 *M* = 3.21 SD = .97	*M* = 1.20 SD = 1.02 *M* = 2.22 SD = .94	*M* = 2.71 SD = 1.07 *M* = 2.92 SD = 1.25

*Note*: *<.05, **<.01, ***<.001. The lower‐left diagonal and the first descriptive row refer to Study 1, whereas the upper‐right diagonal and the second descriptive row refer to Study 2.

## GENERAL DISCUSSION

The current research examined whether reciprocal gifting, conceptualized as an indirect intergroup contact intervention rooted in Mauss' (1967) anthropological studies, could possibly improve Turkish local children's attitudes towards their Syrian refugee peers in classroom contexts characterized by intense prejudice. Study 1 demonstrated short‐term positive changes in attitudes among children who engaged in the original reciprocal gifting procedure; however, these improvements diminished entirely by the follow‐up stage, with no significant changes observed for negative attitudes and social closeness. Addressing the limitations identified in Study 1, Study 2 tested a more comprehensive and enhanced version of the original reciprocal gifting procedure that emphasized participant agency and symbolic engagement. Although this enhanced version resulted in stronger short‐term gains, its effects similarly dissipated by the follow‐up. Consistent across both studies, negative attitudes remained largely unaffected.

The findings from both studies underscore the efficacy of reciprocal gifting in generating immediate positive shifts in attitudes and perceived social closeness. Such short‐term effects align well with prior findings from indirect contact literature investigating imagined and parasocial contact (Crisp & Turner, 2009; Schiappa et al., 2005), highlighting that carefully designed symbolic interactions can meaningfully, albeit temporarily, challenge existing stereotypes and prejudices. The more pronounced initial effect observed in Study 2, where participants actively created personalized gifts, further supports theoretical propositions regarding the importance of agency, symbolic engagement and perceived intentionality in reciprocity‐driven contact (Perugini et al., 2003; Üzümçeker & Akfırat, 2024).

Nevertheless, the rapid attenuation of these effects and the lack of substantial changes in negative attitudes underscore a critical limitation of one‐time, isolated interventions. Research has consistently demonstrated that attitudes, particularly those embedded within long‐standing social categories, are resistant to permanent alteration through single interactions (Hodson & Hewstone, 2013; Saguy, 2018). Sustainable prejudice reduction typically requires repeated exposure and reinforcement (Vezzali & Giovannini, 2012). As such, despite the promising short‐term results, our findings reflect a common pattern within intergroup contact research: Immediate improvements often regress to baseline without ongoing support. However, given that in many cases, we observed positive attitudinal responses to decline over time in all groups, as also observed in the previous studies conducted in the same setting (Bagci et al., 2023), both the original and the enhanced procedures seemed to effectively buffer the ongoing negativity immediately after the manipulation.

An essential consideration in interpreting the current findings also relates to the broader sociopolitical context in which the studies were conducted, especially during Study 1 data collection, which overlapped significantly with Türkiye's 2023 presidential election, a period marked by escalating anti‐refugee rhetoric in public discourse and media (Oxford Analytica, 2023; Korkut et al., 2023; Türkmen, 2023). Even young children, such as those participating in this research, are sensitive to societal narratives transmitted through media, educators and family members (Schachner et al., 2015; Stefanek et al., 2015). Such widespread negative messaging likely exacerbated underlying prejudices, making positive shifts more difficult to maintain. Indeed, the observed trend of declining positive attitudes and increasing negative attitudes in all conditions (in line with Bagci et al., 2023) may reflect the pervasive and influential nature of external socialization processes, which overshadowed the potential long‐term efficacy of our interventions.

At the applied level, our findings provide valuable insights for educators, policymakers and researchers aiming to design effective prejudice‐reduction interventions. Our findings suggest that symbolic reciprocal exchanges can indeed foster short‐term positive interactions, offering a practical approach to initiating attitudinal shifts. However, the observed rapid decay in these effects points to the critical importance of sustained and repeated interventions. Such findings also suggest that this brief period, when attitudes become most open to positive changes, although temporarily, may be an appropriate time whereby further direct or indirect contact strategies could be integrated into the original procedure. As such, imagined contact researchers have argued that imagined contact most likely prepares individuals for actual contact experiences (Crisp & Turner, 2009). Other studies highlighted the superiority of combined procedures involving both direct and indirect contact strategies in their effectiveness (Vezzali et al., 2015), which indicates that the reciprocal gifting procedure could prepare a convenient intergroup setting for the application of further structured contact strategies. Schools may therefore target incorporating micro‐cycle recurring intergroup contact opportunities rather than one‐off events, ideally embedded within broader, continuous multi‐cultural education frameworks (Bigler & Liben, 2007; Rutland & Killen, 2015).

The limited longevity of our intervention's effects underscores the challenge of creating enduring change within a complex sociopolitical ecosystem, where pervasive negative rhetoric and deep‐seated prejudice (Korkut et al., 2023) can easily overshadow a one‐off school‐based initiative. This highlights the necessity for sustained, *multi‐faceted* efforts rather than relying on a *single solution* (Rutland & Killen, 2015). Notwithstanding this challenge, the short‐term improvements we observed suggest that reciprocal gifting may function as a valuable catalyst by activating a unique set of psychological mechanisms. Crucially, receiving a tangible gift from the outgroup likely fosters positive contact meta‐perceptions (Matera et al., 2024), the belief that outgroup members harbour benevolent intentions and are open to positive relations. This concrete signal of goodwill may, in turn, reduce intergroup anxiety and increase willingness for future contact (Crisp & Turner, 2009), a key outcome of indirect contact strategies that prepares individuals for direct encounters. Furthermore, the reciprocity norm (Gouldner, 1960) engendered by the exchange transforms a simple transaction into a meaningful social bond, while the personal investment required in creating a return gift (as in Study 2) may deepen this engagement through effort justification. Thus, while not a panacea, reciprocal gifting can be a potent initial step that makes the outgroup's friendly intentions visibly concrete and thereby opens the door to future, more direct interactions.

Furthermore, the act of giving, especially when it involved personal investment, as in Study 2, where children created artworks on marble canvases, made the intention to build a relationship tangible. This process potentially fostered a sense of symbolic connection and personalization (Brewer & Miller, 1984). By investing their own time, creativity and effort into designing a unique gift, the children were not just sending an object but a part of themselves. This act helps to break down the monolithic *Syrian refugee* category by associating it with a personalized, creative output, thereby facilitating a more individualized and humanized perception of the outgroup.

Adding to this, the enjoyable and fun nature of the gifting activity itself, the excitement of receiving a surprise, the creative freedom in wrapping or painting and the positive social atmosphere may have infused the intergroup concept with positive affect (Paolini et al., 2018). When the concept of *Syrian peers* becomes cognitively linked with the joy and fun of the gifting event, it is likely to create a temporary but significant positive associative shift in attitudes. Finally, the fact that these effects on social closeness were more pronounced in the enhanced condition powerfully underscores the role of self‐engagement and symbolic investment (Aronson & Mills, 1959). The greater effort and personal agency required to create the marble canvases likely deepened the children's psychological investment in the interaction. This heightened engagement may strengthen the internalization of the positive experience, making the symbolic connection to the outgroup more robust and impactful compared with the simpler, less involved repackaging task of Study 1.

### Broader potential of reciprocal gifting

The findings of the present research indicate that reciprocal gifting can produce a short‐term improvement in attitudes towards the outgroup, but that this effect attenuates rapidly over time, and that negative attitudes in particular appear more resistant to change. This pattern may be linked to the nature of the psychological pathways activated by the procedure. In children's everyday lives, reciprocal gifting may mobilize positively oriented processes such as positive affect, gratitude and a sense of closeness (Gim & Harwood, 2024). Moreover, the procedure is not merely an exposure‐based experience. Children physically receive and open a tangible gift and, under a reciprocity obligation, prepare a return gift, thereby enacting a prosocial behaviour towards the outgroup themselves. Accordingly, the impact of the intervention may not be limited to changing perceptions of the outgroup; it may also have the potential to strengthen children's experience of prosocial agency at the self‐level (e.g. *I was able to do something* or *I contributed*) and to foster a positive normative climate within the ingroup (e.g. *This is how we behave* or *This is what we do*). In particular, the personalized and symbolic production component in Study 2 (e.g. creating drawings and messages) may have provided a context that more strongly activates such self‐ and ingroup‐level processes by making effort and self‐expression more salient.

Of course, these possibilities should be treated as *speculative* because they were not directly measured in the current studies. Nonetheless, the active‐participation structure of reciprocal gifting suggests a promising agenda for future research. For example, subsequent studies could test, as mechanisms, post‐gifting gratitude and positive affect, self‐perceptions, perceived ingroup norms and behavioural intentions to support the outgroup. The short‐lived *window of openness* suggested by our findings also raises the question of whether this procedure could yield more durable outcomes when integrated with more intensive and repeated contact‐based strategies rather than being implemented as a one‐off intervention. From this perspective, reciprocal gifting may function as a safe first step that facilitates subsequent contact trajectories in hostile contexts or settings where contact is normatively discouraged and as a starting point in children's social worlds from which collective orientations towards supporting the outgroup may begin to emerge.

### Limitations and future directions

While providing valuable insights, our studies have certain limitations. First of all, methodologically, although we borrowed the reciprocal gifting procedure from anthropological studies, the procedure has been only rarely tested in social psychological research, and thereby, it is largely unknown whether reciprocal gifting should involve an actual or hypothetical interaction in theory. Due to practical reasons, it was not possible to create an intergroup setting whereby children would naturally exchange gifts based on groups. Although we intended to ensure a natural setting for the application of the procedure, we did not assess specifically whether children found the hypothetical gifting process as realistic, which might have created structure but also artificiality. Further studies may design and test whether reciprocal gifting in an actual face‐to‐face interaction would provide more effective outcomes.

Second, our studies lacked the perspectives of teachers and parents, which may profoundly modulate the effectiveness of school‐based intergroup interventions (Schachner et al., 2016; Stefanek et al., 2012). Given the heightened negativity surrounding refugee populations during the election cycle and the overall negative attitudes originating from teachers and parents, it is plausible that indirect messages from adults to children about refugees might have contributed significantly to the limited stability of the intervention effects. In fact, studies in school contexts with refugee children in Türkiye demonstrate that teachers may struggle in managing the integrity of Syrian students in their classrooms and are influenced heavily by the widely held negative stereotypes (Çelik & İçduygu, 2019). Future research must therefore carefully consider these broader ecological and contextual factors when designing interventions intended to produce enduring attitudinal change and involve parents and teachers as critical socialization agents that could contribute to the effectiveness of school‐based programmes.

Third, the effects of intergroup contact might be sensitive not only to the content of the encounter but also to contextual norms and authority support. As highlighted in Allport's (1954) classical formulation, explicit endorsement of inclusive norms by institutions and adult authorities can enhance the effectiveness of contact. In our setting, the presence of teachers and researchers may have increased the salience of injunctive norms favouring inclusion. While we standardized authority presence across all conditions and ensured participation was voluntary, we cannot rule out that children's participation in the gifting task was partly motivated by social compliance with these perceived norms. Such normative signals could have strengthened positive attitudes and closeness in the short term, yet the lack of longevity in our effects suggests that this norm‐based compliance may not have been fully internalized (see Paluck & Green, 2009; Turner & Cameron, 2016). Future research could benefit from testing the intervention under different authority conditions or measuring perceived norms directly to better disentangle their role.

A further important limitation of our study concerns the potential effort imbalance in the reciprocal gifting procedure. In the enhanced procedure of Study 2, children spent significant personal effort in preparing their gifts; however, the gifts they received were standardized pre‐prepared packages. Hence, children perceiving this imbalance in effort may believe less in the outgroup members' goodwill and sincerity (Mauss, 1967). Future studies could control for the level of effort in the exchange or strengthen the perception of matched effort.

Moreover, our gift exchange procedure followed a fixed *receive‐then‐give* sequence. This order may primarily activate a norm of gratitude and reciprocity (Gouldner, 1960). However, reversing the order, which means asking participants to take the first step with a *leap of faith* that the act will be reciprocated, may engage different psychological mechanisms (Yamagishi & Yamagishi, 1994). A *give‐first* approach, by activating proactive trust and cognitive dissonance, could potentially lead to more internalized and durable attitude changes. Future research could experimentally compare these different gifting sequences to determine which approach is more beneficial under varying contextual conditions.

Finally, our research was conducted in a rare intergroup context marked by intense intergroup tension and hostility during a unique sociopolitical period, which may partially explain why negative attitudes were so persistent over time and why positive changes were only temporary. Therefore, current findings may not be generalizable to other intergroup contexts whereby outgroup attitudes are less hostile. A related concern was the exclusion of Syrian children in the interventions due to practical reasons (such as low numbers, language barrier, etc.); ideally, this group would have benefited from the same procedure even more, not only in terms of improved intergroup attitudes, but also potentially in terms of ‘feeling included’. Future research may investigate similar procedures among minority status group members and investigate the broader social psychological effects created by the procedure.

## CONCLUSIONS

In conclusion, this research, for the first time, tested reciprocal gifting as a potential indirect contact strategy that could improve outgroup attitudes in a classroom environment, highlighting how creative, fun and symbolic contact interventions might be used as critical tools to achieve intergroup harmony. While our findings confirmed that reciprocal gift sharing can generate meaningful short‐term changes, they also indicate the considerable challenge of sustaining these improvements over time. Moving forward, efforts to promote durable intergroup harmony must incorporate repeated exposure, engage broader socialization agents and carefully account for contextual influences.

## AUTHOR CONTRIBUTIONS

**Sami Çoksan:** Conceptualization; investigation; funding acquisition; writing – original draft; methodology; validation; visualization; writing – review and editing; project administration; formal analysis; supervision; resources; data curation. **Mustafa Tercan:** Conceptualization; investigation; funding acquisition; methodology; project administration; supervision; resources. **Sabahat Çiğdem Bağcı:** Conceptualization; investigation; writing – original draft; validation; writing – review and editing; methodology. **Serpil Yıldız‐Çoksan:** Conceptualization; investigation; funding acquisition; project administration; resources.

## CONFLICT OF INTEREST STATEMENT

The authors declare that there is no conflict of interest.

## Supporting information


Data S1.

## Data Availability

The dataset, other visual stimuli, measurements and appendices referenced in the text are available at the following OSF link: https://osf.io/768gt/?view_only=5bffa58efe74487490c15786f5ef11da.

## Footnotes

^1^
Mauchly's tests indicated that the assumption of sphericity was not violated for all models, and thus, no corrections were applied.

^2^
The analysis results showing that there was no difference between the groups in terms of pre‐tests for both studies are given in Supplementary‐V.

^3^
Assumption of sphericity was not violated for the models.
