# Supplementary material for: Small gifts, big shifts? Testing the role of contact through reciprocal gifting as a prejudice reduction strategy
Source: Br J Soc Psychol. 2026 Feb 1;65(2):e70052. doi: 10.1111/bjso.70052 (PMC12862200; doi:10.1111/bjso.70052)
Supplement: Supplementary file 1 — Data S1. [file BJSO-65-0-s001.docx]

**Supplementary Materials**

**Supplementary-I. Conceptual Justification for Treating Positive-Negative Attributes and Social Distance as Distinct Constructs**

There were several reasons why we treated positive and negative attributes as distinct constructs. First, the structure of the measurement tool was similar to that used in the original study we cited. Second, across multiple time points in both Study 1 and Study 2, according to the CFAs’ results, we consistently observed that the scale exhibited a two-factor structure. Third, the correlations between average scores for positive and negative attributes at different time points were not high enough to suggest they reflected a single underlying construct. For instance, some correlations were as low as .20 or .29.

Moreover, although relatively older, some empirical studies suggest that individuals can report high or low levels of both positive and negative attitudes simultaneously, even when these appear to be opposites. We employed multiple measures focusing on different yet related components of outgroup evaluation. This approach reflects the multidimensional nature of attitudes, which encompasses cognitive, emotional, and behavioral components (Bohner & Dickel, 2011; Eagly & Chaiken, 1993). Within this framework, the adjective lists were used to capture cognitive and affective evaluations of the outgroup (e.g., kind, rude), while the Bogardus (1925) Social Distance Scale was designed to assess behavioral intentions (e.g., being happy to be in the same class). Furthermore, by measuring positive and negative attitudes as separate subscales, we accounted for the phenomenon of positive-negative asymmetry, often observed in the literature, which suggests that positive and negative evaluations can operate independently (Cacioppo & Berntson, 1994; Pettigrew & Meertens, 1995). Although we hypothesized that our intervention would have a positive effect on all these components, retaining these distinctions allowed us to detect potential differential patterns of effects (for instance, the greater resistance to change of negative attitudes). For all these reasons, we chose to conceptualize positive and negative adjective attributions as indicators of positive and negative attitudes, respectively.

**Supplementary-II: Effects of Reciprocal Gifting on the Control Group After the Follow-Up Phase in Study 1**

For ethical reasons, we implemented the reciprocal gifting procedure for the control group after the follow-up phase in Study 1, measured the outcome, and found that the results followed the same pattern. Positive attitudes mean (*M* = 2.56, *SE* = .16) was higher than only that of the follow-up (*M* = 2.22, *SE* = .16), *t*(51) = 3.62, *p* < .001). No significant effect of the reciprocal gifting procedure was observed for attitudes. Finally, after the reciprocal gifting procedure (*M* = 3.10, *SE* = .13), social closeness increased compared to the follow-up measurement (*M* = 2.49, *SE* = .14; *t*(51) = 4.29, *p* < .001). Positive adjective list’s reliability was α_after follow-up_ = .94, and negative adjective list’s reliability was α_after follow-up_ =.89. Social Distance Scale’s reliability was α_after follow-up_= .84.

**Supplementary-III: Sensitivity Analyses for Significant Results of Study 1 and Study 2**

In Study 1, the effect size for the significant within x between interaction on positive attitudes was η² = .016. First, we converted this value to Cohen’s f and found that it corresponded to an effect size of *f* = .13. We then computed the average of the correlations between the repeated measurements of the relevant variable (pre-test with post-test, post-test with follow-up, and pre-test with follow-up) to obtain an estimate of the within-subject correlation. Using this estimate, we conducted a sensitivity analysis and found that, with an alpha level of .05, 80% power, and the given sample size, we would be able to detect effects as small as *f* = .12. From this perspective, there was no power concern for this analysis.

We repeated the same procedure for social closeness in Study 1. In this case, the effect size was η² = .008, corresponding to *f* = .09. The sensitivity analysis indicated that the smallest effect size we could detect was *f* = .09. Thus, there was no power concern for this analysis either.

We then repeated the same analysis for positive attitudes in Study 2. The interaction effect size was η² = .056, which corresponded to *f* = .24. The sensitivity analysis showed that we would be able to detect effects as small as *f* = .10. For the model with social closeness as the dependent variable in the same study, the observed effect size was *f* = .40, and the sensitivity analysis indicated that we could detect effects as small as *f* = .09. Overall, these results suggest that our analyses for these dependent variables were not underpowered.

**Supplementary-IV: Audio Recording Transcriptions and Images Used in Study 1 and Study 2**

**Audio Transcription of the Male Child (in English)**

Hello friends. My name is Ahmet. I am a Syrian immigrant student, and like you, I am in the 4th grade. I have been studying in Türkiye for four years, and I hope my Turkish is good enough. First of all, thank you all for accepting us into your classroom. Actually, Fatma and I would have liked to visit you in person, but since it is the school term, we could not come. Instead, we are sending you the gifts that were prepared by me, Fatma, and our Syrian classmates. We hope you like them. Now, Fatma will speak.

**Audio Transcription of the Female Child (in English)**

Hello friends, my name is Fatma. Like Ahmet, I am also a Syrian immigrant student, and I am in the fourth grade. As a class, we prepared some gifts for you to get to know you and become friends. I hope you like them. We selected these gifts together as Syrian students in our class. Thank you once again for accepting our presents.

**AI-Generated Visual of a Syrian Male Child**


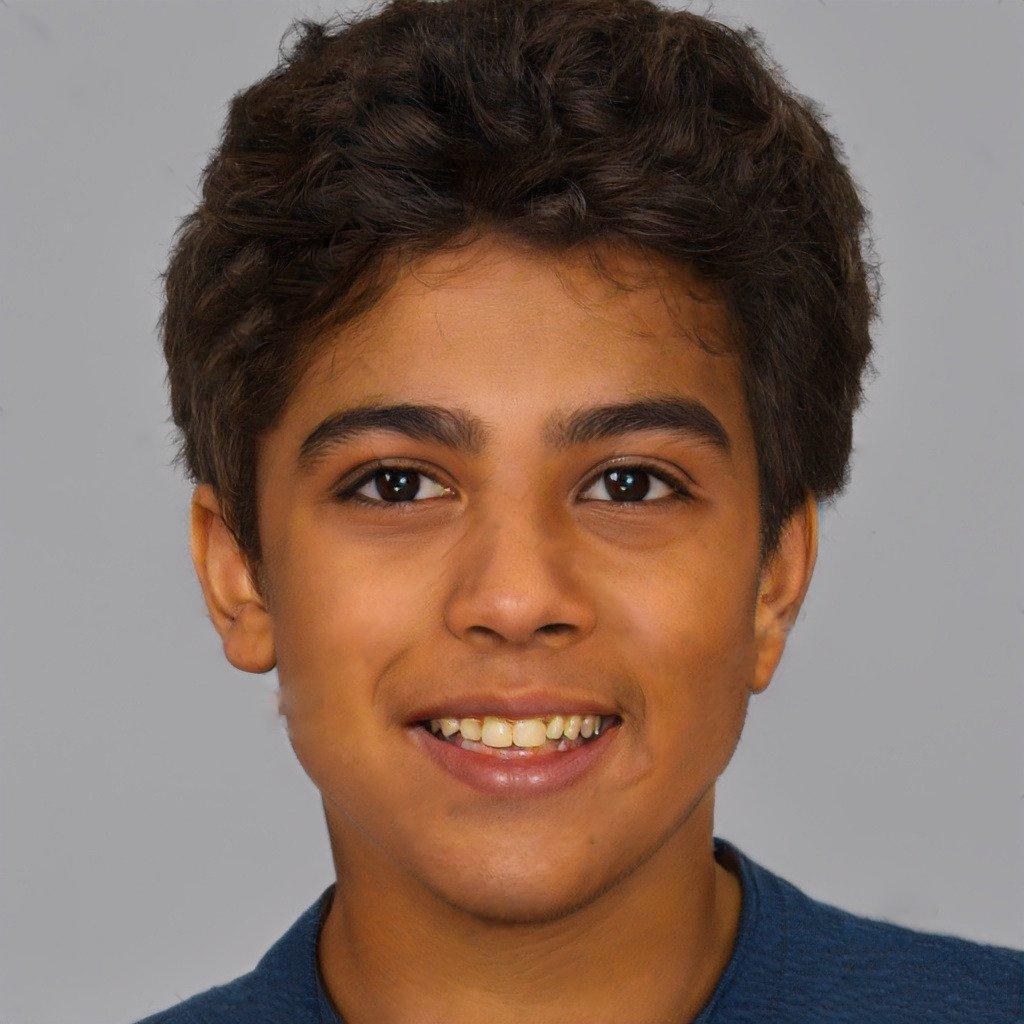


**AI-Generated Visual of a Syrian Female Child**


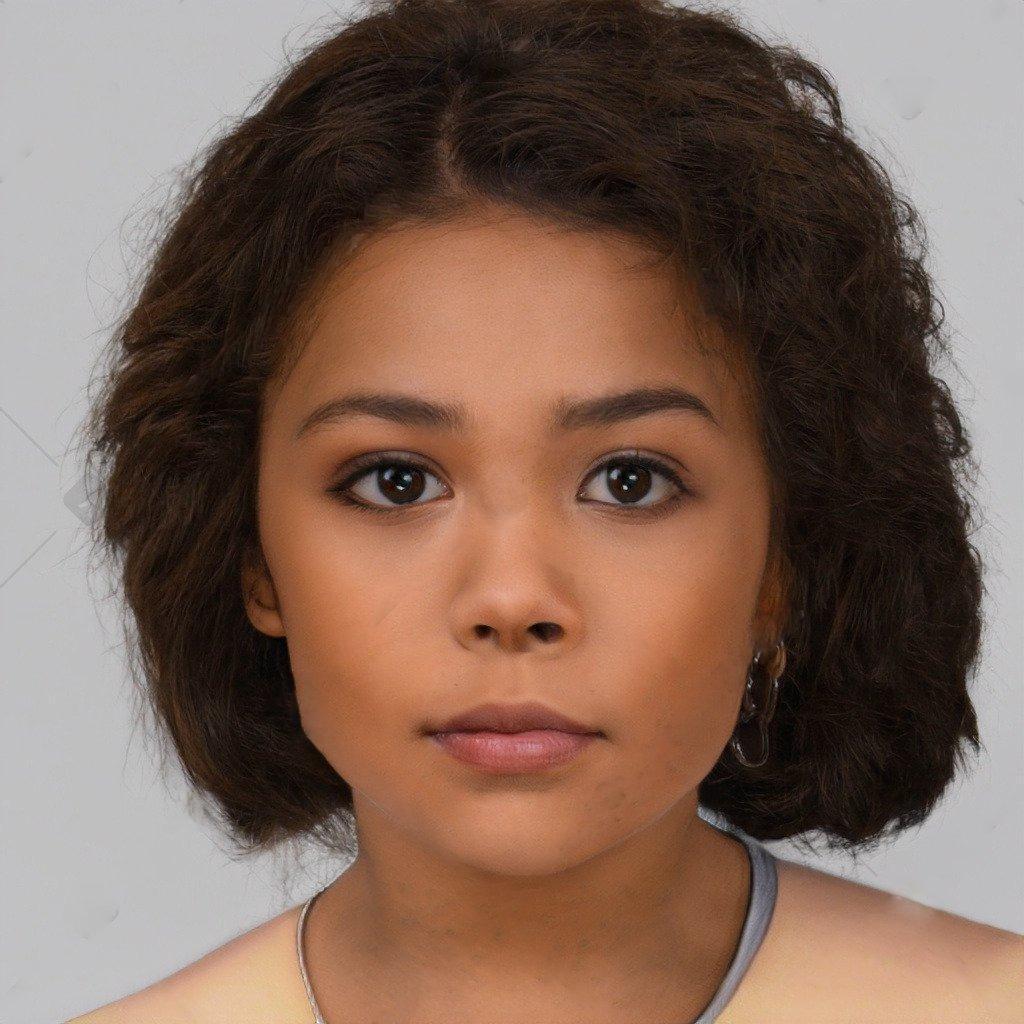


**Supplementary-V: Equivalence of Groups at Baseline (Pre-test)**

ANOVAs for the outcomes revealed no differences between groups at baseline in terms of outcomes. In Study 1, there was no difference between the experimental (*M* = 2.61, *SE* = .09) and the control group (*M* = 2.78, *SE* = .12) in terms of positive attribution, *t*(142) = 1.10, *p* = .272. Also, there were no differences between groups in terms of negative attribution (*M_experimental_* = .82, *SE_experimental_* = .09; *M_control_* = .95, *SE_control_* = .11; *t*(142) = .93, *p* = .356) and social closeness (*M_experimental_* = 3.38, *SE_experimental_* = .09; *M_control_* = 3.41, *SE_control_* = .12; *t*(142) = .23, *p* = .821).

In Study 2, post hoc comparisons of the groups showed that there was no difference between the enhanced reciprocal gifting group (*M* = 3.27, *SE* = .12) and the reciprocal gifting group (*M* = 3.17, *SE* = .10; *t*(204) = .62, *p* = .536), the reciprocal gifting group and the control group (*M* = 3.41, *SE* = .10; *t*(204) = 1.73, *p* = .085), and the enhanced reciprocal gifting group and the control group (*t*(204) = .88, *p* = .378) in terms of positive attribution. Moreover, in terms of negative attributions, there was no difference between the enhanced reciprocal gifting group (*M* = 2.11, *SE* = .12) and the reciprocal gifting group (*M* = 2.15, *SE* = .09; *t*(204) = .25, *p* = .799), the reciprocal gifting group and the control group (*M* = 2.02, *SE* = .09; *t*(204) = 1.02, *p* = .310), and the enhanced reciprocal gifting group and the control group (*t*(204) = .63, *p* = .530). Furthermore, in terms of social closeness, there was no difference between the enhanced reciprocal gifting group (*M* = 2.81, *SE* = .16) and the reciprocal gifting group (*M* = 3.15, *SE* = .13; *t*(204) = 1.66, *p* = .099), and the reciprocal gifting group and the control group (*M* = 3.24, *SE* = .13; *t*(204) = .50, *p* = .616). However, at the baseline (i.e., pre-test), the control group had greater social closeness than the enhanced reciprocal gifting group, *t*(204) = 2.10, *p* = .037. As a result, in both Study 1 and Study 2, all groups appear to be at the same level across all outcomes at baseline, that is, in the pre-tests. Only in Study 2 does the control group show higher social closeness than the enhanced reciprocal gifting group.

**Supplementary-VI: Effects of Reciprocal Gifting on the Control Group After the Follow-Up Phase in Study 2**

For ethical reasons, we administered the new reciprocal gifting procedure to participants in the control group at the end of the study and collected outcome measures in Study 2. The results of mixed-design repeated measures ANOVAs conducted for each outcome indicated the same pattern. There was a declining trend in positive attitudes and social closeness and an increasing trend in negative attitudes prior to the intervention. Following the new reciprocal gifting procedure, participants’ positive attitudes toward their Syrian peers (*M* = 3.47, *SE* = .10) and social closeness (*M* = 3.23, *SE* = .14) significantly increased compared to their previous measurements (*M* = 3.20, *SE* = .10; *M* = 2.93, *SE* = .14, respectively; *t*(78) = -3.114, *p* = .013; *t*(76) = -3.300, *p* = .008, respectively), while negative attitudes (*M* = 2.06, *SE* = .09) significantly decreased compared to their prior scores (*M* = 2.28, *SE* = .10; *t*(78) = 2.874, *p* = .005). Positive adjective list’s reliability was α_after follow-up_ = .89, and negative adjective list’s reliability was α_after follow-up_ =.89. Social Distance Scale’s reliability was α_after follow-up_= .89.

**Supplementary-VII: Tests for the Moderating Role of Baseline Attitudes**

In Study 1, we conducted simple moderation analyses by treating the pre-test scores of positive attribution, negative attribution, and social closeness as moderators to examine the effect of participants’ group membership (experimental vs. control) on the corresponding outcome’s post-test or follow-up measurements. In these six models, standard errors were estimated using the bootstrap method (1,000 resamples), and standard coding (0 and 1) was applied for the groups. None of these models revealed a significant moderating effect.

In Study 2, we repeated the same procedure. This time, while focusing on the same moderator–outcome relationships, we examined the predictive role of group membership in pairwise comparisons (enhanced reciprocal gifting vs. reciprocal gifting; enhanced reciprocal gifting vs. control; reciprocal gifting vs. control). Across these 18 models, the pre-test score of the corresponding outcome did not show any significant moderating effect.^^[[1]](#footnote-1)^^

**Supplementary References**

Bohner, G., & Dickel, N. (2011). Attitudes and attitude change. *Annual Review of Psychology, 62*, 391-417. <https://doi.org/10.1146/annurev.psych.121208.131609>

Cacioppo, J. T., & Berntson, G. G. (1994). Relationship between attitudes and evaluative space: A critical review, with emphasis on the separability of positive and negative substrates. *Psychological Bulletin, 115*(3), 401-423. <https://doi.org/10.1037/0033-2909.115.3.401>

Eagly, A. H., & Chaiken, S. (1993). *The psychology of attitudes.* Harcourt Brace Jovanovich College Publishers.

Pettigrew, T. F., & Meertens, R. W. (1995). Subtle and blatant prejudice in western Europe. *European Journal of Social Psychology, 25*(1), 57–75. <https://doi.org/10.1002/ejsp.2420250106>

1. Due to the nature of the bootstrap method, p-values may show slight variations. Some significance levels are around .05. Although in certain models the value may fall below .05, and a correction should be applied since the same variables were tested multiple times. Hence, readers should take this point into consideration. [↑](#footnote-ref-1)
